# Enhanced Energy Absorption and Flexural Performance of 3D Printed Sandwich Panels Using Slicer-Generated Interlocking Interfaces

**DOI:** 10.3390/polym18010094

**Published:** 2025-12-29

**Authors:** Amged Elhassan, Hour Alhefeiti, Mdimouna Al Karbi, Fatima Alseiari, Rawan Alshehhi, Waleed Ahmed, Al H. Al-Marzouqi, Noura Al-Mazrouei

**Affiliations:** 1 Mechanical and Aerospace Engineering Department, UAE University, Al Ain 15551, United Arab Emirates; 2 Chemical and Petroleum Engineering Department, UAE University, Al Ain 15551, United Arab Emirates; 3 Engineering Requirements Unit, UAE University, Al Ain 15551, United Arab Emirates

**Keywords:** 3D printing, composite, flexural, interlock, sandwich

## Abstract

This study assessed the effect of slicer-made interlocking joints on 3D printed sandwich panels manufactured through fused filament fabrication (FFF) in terms of flexural properties and energy absorption. Composites were prepared with thermoplastic polyurethane (TPU) as the core material and polyamide (PA), polylactic acid (PLA), polyethylene terephthalate (PET) as skin materials for each of the three composites, respectively. In order to assess the implications of internal geometry, 3D printing was done on five infill topologies (Cross-3D, Grid, Gyroid, Line and Honeycomb) at 20% density. All samples had 20% core density and underwent three point bending testing for flexural testing. It was noted that the Grid and Gyroid cores had the best performance in terms of maximum load capacity based on stretch-dominated behavior while Cross-3D and Honeycomb had lower strengths but stable moments during the bending process. Since Cross-3D topology offered the lowest deflection, it was selected for further experiments with slicer added interlocks at the face–core interface. This study revealed the most notable improvements as gains of up to 15% in peak load, 48% in maximum deflection, and 51% in energy absorption compared with the non-interlocked designs. The PET/TPU interlocked demonstrated the best performance in terms of the energy absorption (2.45 J/mm^3^) and peak load (272.6 N). In contrast, the PA/TPU interlocked exhibited the best flexibility and ductility with a mid-span deformation of 21.34 mm. These results confirm that slicer-generated interlocking interfaces lead to better load capacity and energy dissipation, providing a lightweight, damage-tolerant design approach for additively manufactured sandwich beams.

## 1. Introduction

There is growing research interest in lightweight and high-efficiency multilayered structural composites produced through fused deposition technology with enhanced energy absorption performance, particularly in aerospace, automotive, and civil engineering applications. Traditional manufacturing procedures imply a standard sandwich panel so that the production of core cell geometries is limited. Moreover, the 3D printed panels produce an entirely one-piece construction, limiting issues of adhesion between parts. Additively manufactured composite structures were superior to conventional composites in three separate findings: 3D printed out of plane and S-shape cores exhibit a 55% increase in flexural strength, a 195% increase in tensile strength, and a 32% increase in energy absorption at reduced relative density [1]. Hybrid AM panels using PEI-Ultem lattices cores outraced polymer foams and rivaled Nomex and aluminum honeycombs in stiffness at 1/3 to 1/4 the weight [2].

Li et al. [3] investigated novel multilayer thin-walled sandwich polymer structures exhibiting remarkable energy absorption and mechanical properties. Their study demonstrated how multilayer configurations notably improve lightweight design and impact resistance, establishing a framework for advanced polymeric sandwich panels. Similarly, Chahardoli and Akhavan Attar [4] combined theoretical and experimental analyses to assess sandwich panels featuring 3D printed cores, revealing the significance of core geometry and thickness optimization in enhancing structural performance and failure resistance. Mirzaei et al. [5] analyzed experimentally 3D printed honeycomb core-reinforced sandwich panels under compression loading; the findings revealed that energy absorption performance strongly depends on core orientation and fiber reinforcement type, indicating that vertically oriented cores and carbon fiber skins yield the highest energy dissipation before densification. Extending this work, Fareed et al. [6] investigated the mechanical response of 3D printed lattice core sandwich structures, emphasizing the critical role of lattice geometry in affecting flexural stiffness and impact behavior. Deng and Liu [7] employed a multi-objective design optimization strategy for thin-walled sandwich tubes incorporating laterally corrugated cores, achieving improved energy dissipation and crash resistance under lateral compressive loads. Grondin et al. [8] examined the bending performance of AM-fabricated sandwich composites with bioinspired functionally graded materials, showing greater flexural rigidity and energy absorption. Hosseinpour et al. [9] studied multilayer ultra-lightweight sandwich panels developed through hybrid additive-electroforming processes. Their work underscored the significant influence of hybrid metallic laminations, which led to notable increases in yield strength and energy dissipation efficiency, pointing to novel hybrid manufacturing approaches for future high-performance sandwich composites. A comprehensive evaluation of sandwich panels with composite and polymeric foam cores was conducted by Mirzaei et al. [10], who identified core crushing and plastic yielding as the primary failure mechanisms controlling overall energy absorption behavior. Further studies conducted by Burlayenko and Sadowski [11], and Bragagnolo et al. [12], investigated interface debonding and dynamic loading responses in foam and honeycomb core sandwich panels, providing critical insights into vibration stability and overall structural integrity. Research by Li et al. [13] on hierarchical honeycomb and auxetic metamaterial configurations expanded the understanding of how structural hierarchy affects static compression and multi-impact energy absorption. Boursier et al. [14] combined hands-on testing with numerical analysis on lattice-configured structures tailored for automotive crash absorption, showing how additive manufacturing facilities can fabricate complex designs that improve impact energy more efficiently under dynamic loading scenarios. In a related study, Geramizadeh et al. [15] explored the influence of face sheet thickness on bending behavior in honeycomb panels made via FDM, determining the key design factors that control load capacity.

In addition, Yazdani Sarvestani examined failure scenarios and multi-impact resistance of 3D printed meta sandwich structures, demonstrating the significance of material selection and geometric configuration on multi-impact absorption behavior, summarizing limitations and potential future research directions, including the integration of innovative materials and data-driven design optimization [16]. Collectively, these findings indicate that optimized parameters of additive-manufactured sandwich configurations by selecting suitable materials, core topology design, and layer arrangements lead to lightweight and high-strength performance. Ongoing interdisciplinary efforts integrating experimental and simulation methods are anticipated to promote the adoption of these novel composites across high-value industrial industries [17].

Despite extensive research on 3D-printed multilayer panels, no peer-reviewed experimental studies have yet examined the effect of slicer-based interlocking features generated via Ultimaker Cura 5.10.2 on the flexural performance and energy absorption of such structures. Kuipers et al. introduced the Interlaced Topologically Interlocking Lattice (ITIL), sliced with the Cura Arachne Engine to fabricate dual-material specimens combining PLA and PP; however, their investigation was restricted to tensile testing [18]. Building on this foundation, the present study employs the Generate Interlocking Structure function in Ultimaker Cura conceptually derived from the ITIL model by Kuipers et al. to experimentally evaluate the flexural response and energy absorption of 3D-printed non-interlocked and interlocked multilayers composed of a thermoplastic polyurethane (TPU) core and different skin materials: polyamide (PA), polylactic acid (PLA), and polyethylene terephthalate (PET). This work represents the first systematic experimental validation of a commercially implemented slicer-generated interlocking function under flexural loading.

This research investigates the effect of slicer-generated interlocking interfaces on the flexural response and energy absorption of 3D-printed sandwich panels produced by fused filament fabrication (FFF). Furthermore, the work also includes a systematic comparison of five infill patterns (Cross-3D, Grid, Gyroid, Line, and Honeycomb) to quantify their influence on flexural response. The study employs thermoplastic polyurethane (TPU) as the core material and three distinct skin materials polyamide (PA), polylactic acid (PLA), and polyethylene terephthalate (PET). All sandwich specimens are fabricated using the Ultimaker Cura slicer with and without the interlocking feature to assess the influence of interfacial design on mechanical performance. The panels are evaluated under three-point bending tests in accordance with ASTM D7250M to determine flexural strength, deformation capacity, and energy absorption. The outcomes of this investigation are expected to provide new insights into the role of slicer-driven interlocking in enhancing the mechanical efficiency and energy dissipation of additively manufactured multilayer composites.

## 2. Methodology

**Figure 1:**
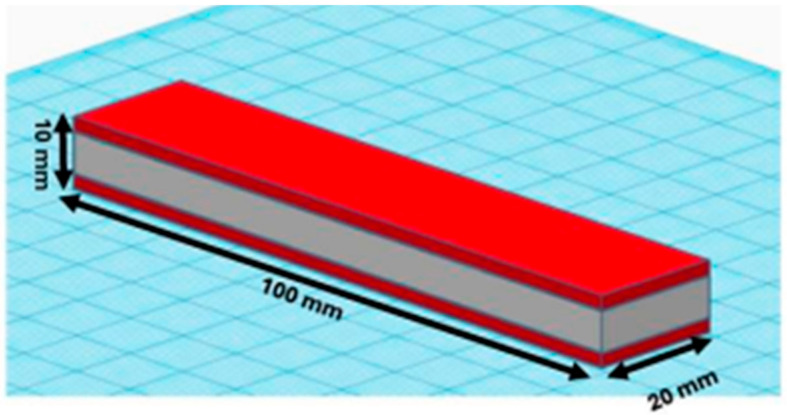
Modeling of the sandwich structure.

**Figure 2:**
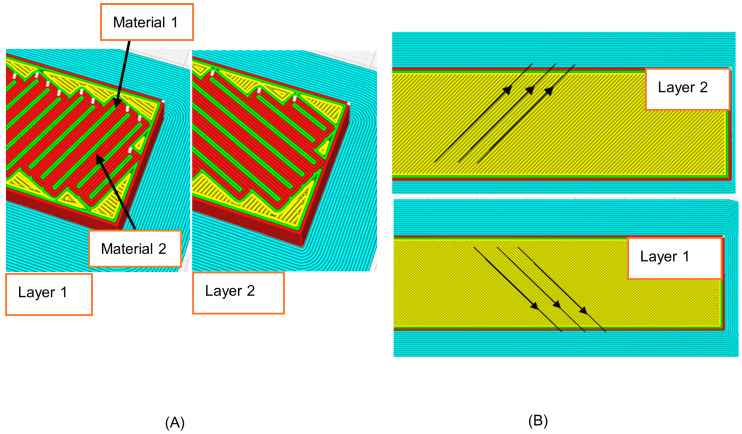
(**A**) Sliced interlocked interface model; (**B**) Non-interlock interface model.

This work utilized a three-point bending setup to assess the flexural performance of 3D printed multilayer composite panels experimentally. Each panel has the same geometry as shown in Figure 1. The core thickness was 6 mm, and the outer skins (2 mm). Three sandwich variants were tested in two configurations, with and without the interlocking feature in Ultimaker Cura (Ultimaker Cura 5.10.2) as shown in Figure 2. The details of the interlock of Figure 2A, which Cura produces, is basically a repeated peg-and-hole structure at the material interface. It essentially interlocks with the layers adjacent to it. This looks like teeth interlocking in cross section, or a comb that goes one way and then perpendicular on the *Z*-axis going the other, making a weave in 3D to support interfacing stability as well as strain distribution. To achieve the interlocking feature at the core–skin interfaces while maintaining 20% infill in the core, solid interface layers were introduced at both the top and bottom of the TPU core. Specifically, each interface consisted of six solid line-pattern layers (total thickness 1.2 mm), within which a two-layer interlocking region with a width of 0.8 mm was generated by the slicer.

**Figure 3:**
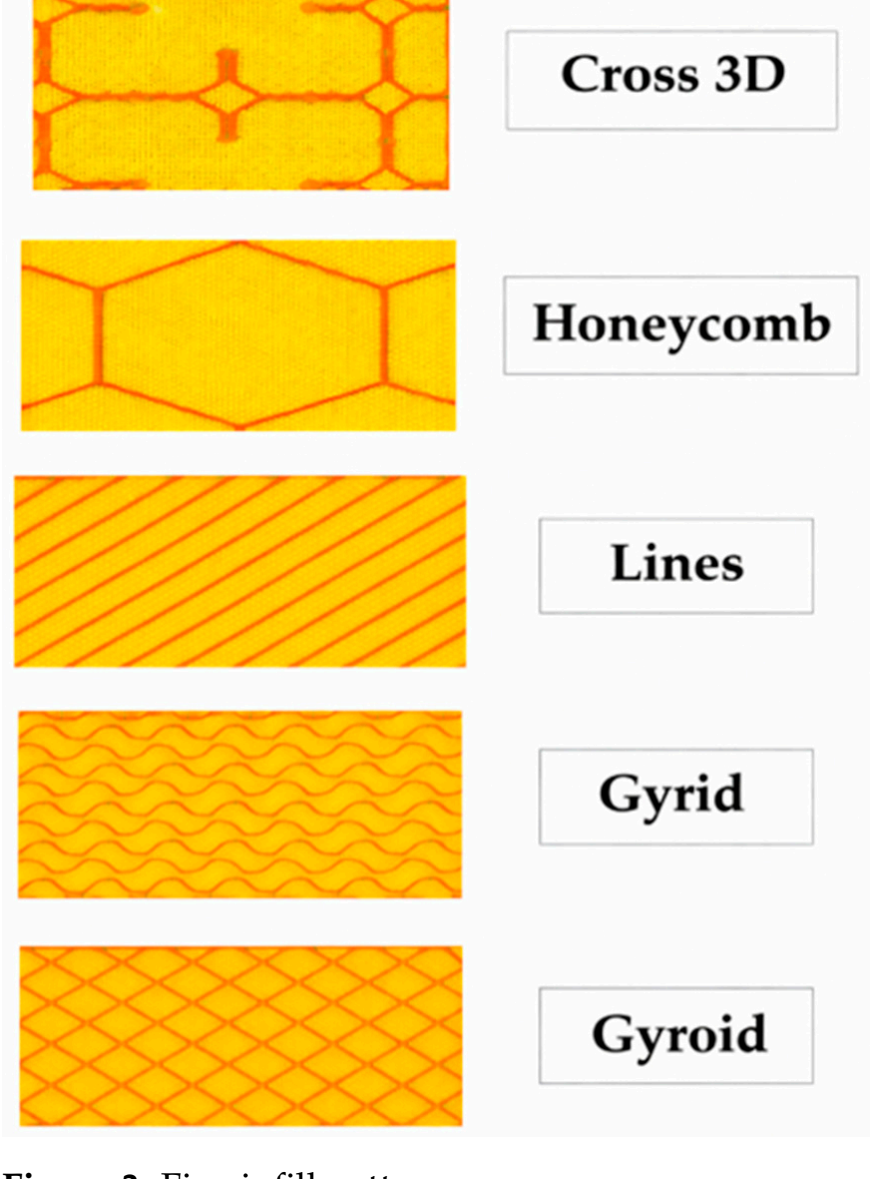
Five infill patterns.

All samples featured a TPU core with varied face sheet materials. Table 1 lists the elastic modulus of each material, where the face sheet values correspond to the solid polymer modulus, while the core values represent the effective elastic modulus calculated for the 20% infill configuration. Fabrication was produced by FFF using five different infill patterns, shown in Figure 3 (Cross 3D, Honeycomb, Line, Grid, and Gyroid), and default Cura slicer settings. Ultimaker UM S5 (UltiMaker B.V., 2018, Watermolenweg 2, 4191 PN Geldermalsen, Gelderland, The Netherlands) has been used in this study to produce the printed samples which has dual nozzles for printing different materials.

Reliability was ensured by performing mechanical testing on multiple 3D printed samples. First, three samples were printed per testing condition using the same print settings and same resin batch. They were all subjected to bending testing under the same conditions. However, during testing, some of the samples broke differently from one another. Some were broken early because of some results or printing induced fractures (layer separation, surface imperfections, or poorly glued layers). Some were broken early due to test-induced fractures (slipping, improper loading). Therefore, some samples had to be reprinted and re-tested to obtain results more conclusive that aligned more closely with the average of the batch as there are numerous induced errors of chance to consider with 3D printing, especially when layer adhesion and surface quality are crucial to mechanical soundness. Thus, the results discussed in this study are based on the most consistent results throughout the testing series as these results were found to be representative of the material and printing settings in focus under the conditions of the experiment for such conditions to yield optimal results based on such settings. This study emphasized the printing parameters to ensure the consistent and dependable fabrication of all specimens, thus maintaining the quality of the printed samples. Table 2 comprehensively summarizes the standard printing parameters utilized for the specimens. All specimens were printed using a dual copper AA nozzle (0.4 mm) with an initial layer height of 0.2 mm, a wall thickness of 0.8 mm (two wall lines, outer wall wipe distance 0.8 mm), line widths of 0.4 mm for walls and infill and 0.5 mm for top/bottom regions, and a fixed top/bottom thickness of 1.2 mm with one top surface skin layer (top surface line width of 0.5 mm for the TPU core and 0.4 mm for the face materials). Material-specific nozzle temperatures were applied: 225 °C for TPU, 205 °C for PLA, 245 °C for PA, and 275 °C for PET to ensure consistent print quality and interlayer adhesion.

**Figure 4:**
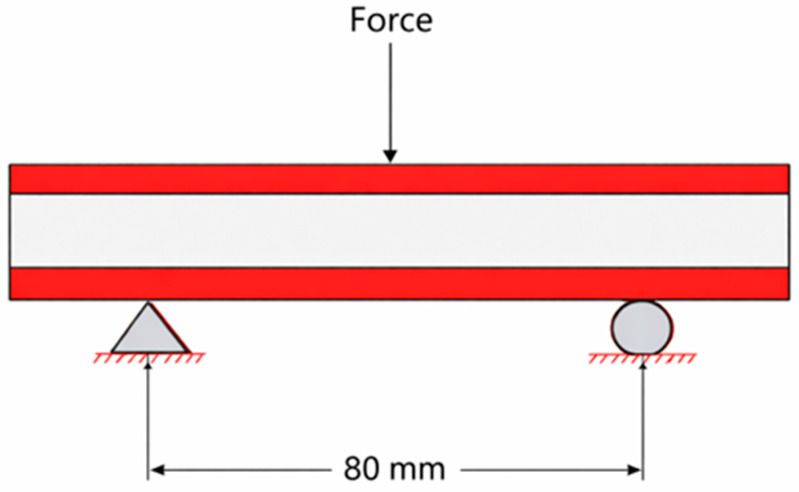
Experimental setup.

An experiment was carried out by subjecting the composite sandwich beam shown in Figure 4 to a bending load. The load applied on the sandwich beam was at the midspan of the beam’s length. The beam is supported on the left by a triangular fixture with Ux = 0 and Uy = 0 boundary conditions, while the right of the beam is supported by a roller fixture with Ux ≠ 0 and Uy = 0 boundary conditions. The load is applied in some way along its length. All specimens were tested using a universal testing machine (Shimadzu, Kyoto, Japan) of the same model (WDW-10) and calibration. Initial trials were conducted with a 100 kN load cell; however, subsequent tests used a 10 kN load cell to achieve higher resolution within the measured load range. This adjustment ensured more precise data acquisition while maintaining full consistency in testing conditions with a cylindrical head test of 5 mm according to the ASTM D7250M standard [20]. For each combination, three samples were printed.

## 3. Finite Element

**Figure 5:**
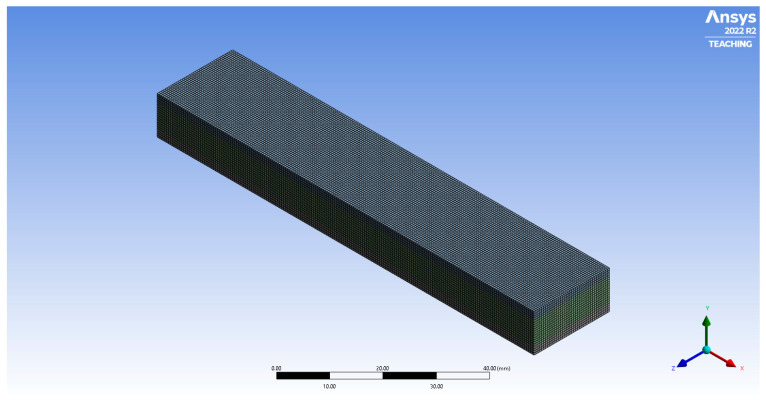
The mesh of the model.

The Finite Element Method (FEM) is a numerical technique for solving problems that are described by partial differential equations or can be formulated as functional minimization [21,22]. Finite element analysis was conducted using ANSYS (2022 R2 (22.2)) to simulate the bending behavior of the three different sandwich panels under the same boundaries conditions as the experimental test. A simplified multilayer sandwich beam model has been adopted as well as elastic shear and peel stresses in an adhesive joint between the faces. Linear elastic properties have been considered in the FEA, since the engineering components are designed with the elastic zone that simplifies the handling of complicated cases. The number of nodes and elements was kept as 67,891 and 160,000, respectively. The face mesh schematic of the model is shown in Figure 5.

## 4. Theoretical Analysis

The elastic modulus (E) of a cellular material (e.g., foam, lattice, or porous solid) is strongly influenced by its relative density $\left(\frac{\rho }{\rho_{s}}\right)$, where ρ $\frac{kg}{m^{3}}$ is the density of the cellular material and $\rho_{s}$ $\frac{kg}{m^{3}}$ is the density of the solid from which it is made [23]. The relationship can be derived using micromechanical scaling laws, considering deformation mechanisms in cellular structures [24,25]. Two primary models exist based on the microstructure: bending-dominated and stretching-dominated behavior [26,27].

Bending-Dominated Cellular Structures (Open-Cell Foams):

For low-density foams where cell walls undergo bending under load, the elastic modulus scales with the square of the relative density [28]. The deflection σ [MPa] of a beam under bending is given by Euler–Bernoulli beam theory:

(1) $$\sigma =\frac{FL^{3}}{3E_{S}I}$$

where F (N) is the applied force, L (m) is the beam length, $E_{S}$ [MPa] is the solid modulus, and *I* ($m^{4}$) is the second moment of area, where $I\propto t^{4}$ for a square coross section of thickness t (m).

The relative density ($\frac{kg}{m^{3}}$) scales with the beam thickness:

(2) $$\frac{\rho }{\rho_{s}}\propto {\left(\frac{t}{L}\right)}^{2}$$

The effective modulus E of the beam is related to the stiffness of the beam network.

(3) E∝FL2σL

(4) $$E\propto \frac{E_{S}I}{L^{4}}$$

It can be written as:

(5) $$\frac{E}{E_{s}}\propto {\left(\frac{\rho }{\rho_{s}}\right)}^{2}$$

For structures where deformation is primarily due to axial stretching/compression (e.g., truss lattices), the modulus scales linearly with relative density, k represent stiffness (N/m)

(6) $$k\propto \frac{E_{s}A}{L}$$

where A (${mm}^{2}$) is the cross-sectional area (A∝ t^2^). The relative density is

(7) $$\frac{\rho }{\rho_{s}}\propto \frac{A}{L^{2}}{\propto (\frac{t}{L})}^{2}$$

The effective modulus E is derived from the stiffness per unit area:

(8) $$E\propto \frac{K}{L}\propto \frac{E_{S}A}{L^{2}}\propto \frac{\rho }{\rho_{s}}$$

For cellular materials, the relationship is often described by the Gibson–Ashby model:

(9) $$\frac{E}{E_{s}}=C{\left(\frac{\rho }{\rho_{s}}\right)}^{n}$$

where *C* is a proportionality constant (depends on microstructure), *n* = 2 for bending-dominated foams, *n* = 1 for stretching-dominated lattices.

In 3D-printed parts with partial infill, the effective modulus scales similarly. For example, honeycomb infill, $E\propto \rho^{2}$ (bending-dominated) and grid infill: E∝ρ (stretching-dominated).

It optimizes the strength-to-weight ratio by adjusting infill density and pattern.

The flexural rigidity (N·$m^{2}$) of a beam is given by

(10) D=EI

For a rectangular cross-section [width B (m), height H (m)]

(11) $$I=\frac{BH^{3}}{12}$$

Flexural rigidity of the Core (EI)core

The core is typically a homogeneous material (e.g., foam) with height H_core_ and modulus E_core_

Using the standard moment of inertia for a rectangle:

(12) $$I=\frac{B{H^{3}}_{core}}{12}$$

(13) $${EI}_{core}=E_{core}\frac{B{H^{3}}_{core}}{12}$$

The flexural rigidity of the face plates ${2(EI)}_{plate}=2(\frac{BH^{3}}{12}+hBr^{2})E_{plate}$.

Where h (m) is the thickness of plate, r (m) is the distance from the neutral axis, $E_{plate}$ is the modulus of elasticity of the plate material.

Total Flexural Rigidity of the Composite (EI)comp:

(14) $${\left(EI\right)}_{comp}={\left(EI\right)}_{core}+{2(EI)}_{plate}\;$$

where ${\left(EI\right)}_{comp}$ is the Total flexural rigidity of the composite.

## 5. Results & Discussion

### 5.1. Non-Interlock Composites Analysis

Three sandwich composites were fabricated using FFF with skins of polylactic acid (PLA), polyethylene terephthalate (PET), and polyamide (PA), each incorporating a TPU core. This section presents an analysis of the mechanical behavior of 3D printed sandwich panels without interlock fabricated with varying infill patterns. The study investigates how changes in TPU infill geometries influence load-bearing capacity, deformation characteristics, and energy absorption. Through a combination of experimental testing, theoretical calculations, and finite element simulations, the results are examined across multiple material combinations to identify trends, evaluate structural efficiency, and draw conclusions relevant to lightweight structural design. The processed data were visualized to reach the conclusions, and comparative analysis was conducted, and a comparison of results was conducted.

#### 5.1.1. PA-TPU-PA

**Figure 6:**
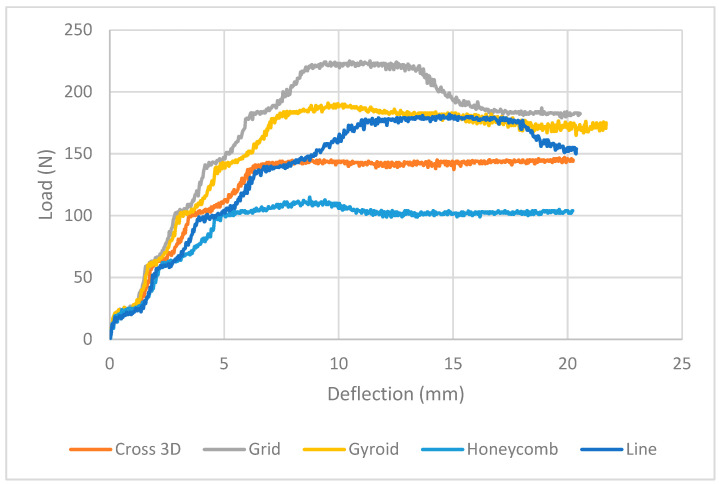
Load vs. Extension PA-TPU-PA (Experimental) Full Zone.

It can be seen in Figure 6 (Table 3) that with PA skins fixed and the core diluted to 20%, the infill geometry sets the response. Grid carries the highest load, peaking ~225 N at ~10 mm and then sitting around 180 N to the end. Line and Gyroid are close behind: both crest near 180–190 N; Line holds a long 175–180 N shoulder with a slight late dip, while Gyroid declines gently to ~175 N. Cross-3D never climbs above ~145 N but is very stable, and Honeycomb is the softest (~100–110 N plateau). Table 3 summarizes the key findings.

**Figure 7:**
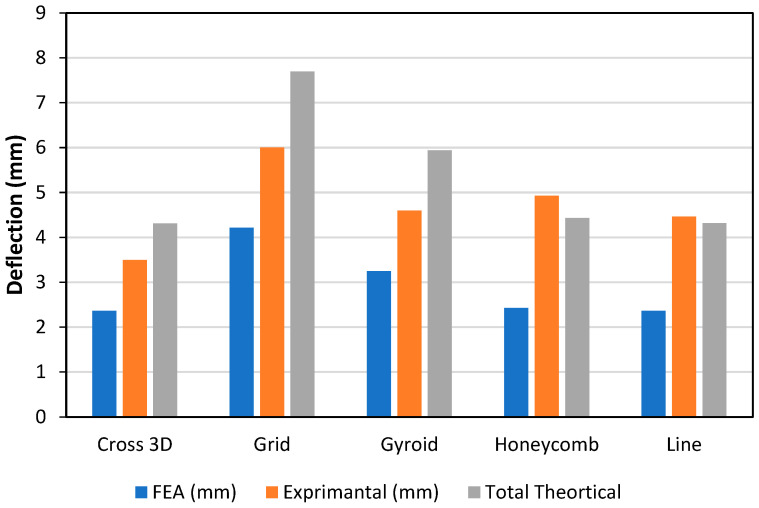
Deflection and Infill Pattern, PA-TPU-PA (Elastic Zone).

Stiffness is driven by the infill as shown in Figure 7 by the experimental bars (lower = stiffer) the order is Cross-3D (~3.5 mm) → Line (~4.4 mm) → Gyroid (~4.6 mm) → Honeycomb (~4.9 mm) → Grid (~6.0 mm), so Grid is the most compliant at this density. FEA is consistently the stiffest prediction (≈25–45% below the tests for most patterns). The closed-form theory is mixed: it is too soft (over-predicts deflection) for Cross-3D, Grid and Gyroid; close for Line; and too stiff for Honeycomb. The gaps track pattern physics homogenized core modulus not matching each lattice, TPU non-linearity, and small skin core slip.

#### 5.1.2. PET-TPU-PET

**Figure 8:**
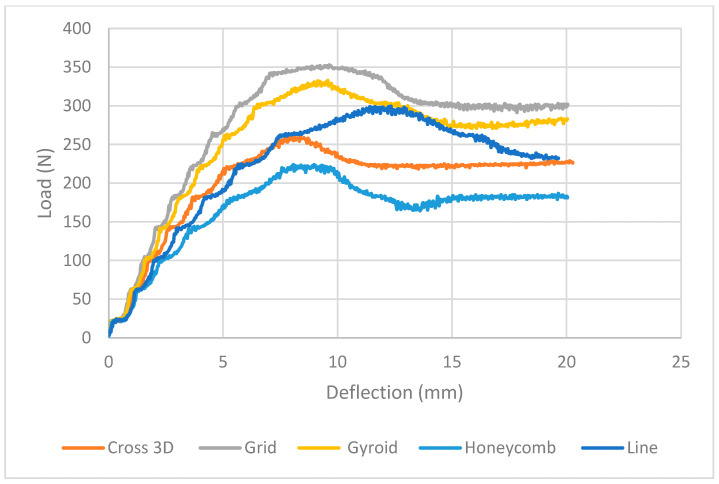
Load vs. Extension PET-TPU-PET, (Experimental) Full Zone.

Keeping the PET face-sheets the same but varying only the core geometry, the load–deflection responses are divided neatly, as illustrated in Figure 8 (Table 4). The stand-out is Grid: it crests at the highest (~350 N at ~9–10 mm) and then falls back to the highest plateau (~295–300 N), providing the greatest energy. Gyroid is second (crest ~325–330 N; very stable tail ~270–280 N). Line ramps up smoothly to ~300–310 N but softens more, ending at ~230–240 N. Cross-3D is similarly in the same mid/low range with a wide shoulder ~235–245 N after an initial peak ~255–265 N. Honeycomb is most compliant: low peak ~220–225 N followed by a low, flat plateau ~175–185 N. No catastrophic sudden drops here (unlike some examples of 40%-core); softening after the peak is consistent with strut buckling and shear in the lower-density TPU core.

**Figure 9:**
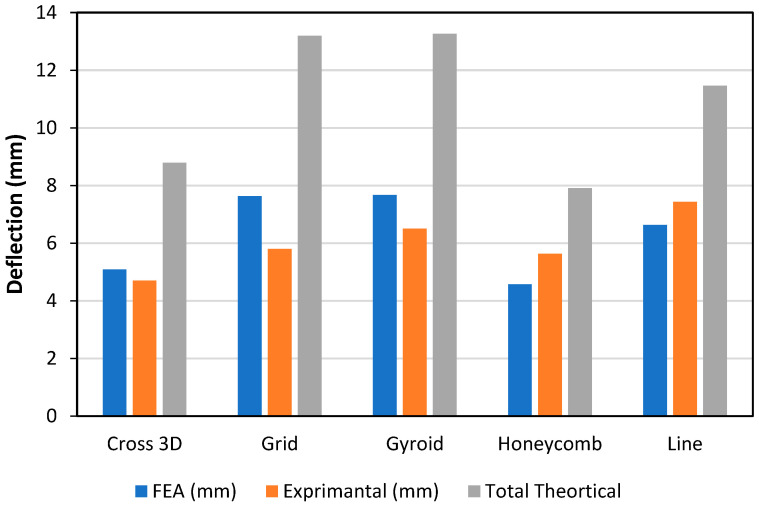
Deflection and Infill Pattern, PET-TPU-PET (Elastic Zone).

As shown in Figure 9, at the same load, the measured deflection (stiffest is the most compliant) is Cross-3D (~4.7 mm) < Honeycomb (~5.6 mm) ≈ Grid (~5.8 mm) < Gyroid (~6.5 mm) < Line (~7.4 mm), so Cross-3D gives the best small-deflection stiffness and Line the least. The FEA follows the ranking but misses magnitudes too soft for Cross-3D/Grid/Gyroid by ~10–30% and too stiff for Honeycomb/Line by ~15–20%. The classical sandwich theory is consistently too compliant, especially for Grid/Gyroid/Line (often 50–120% over-prediction).

#### 5.1.3. PLA-TPU-PLA

**Figure 10:**
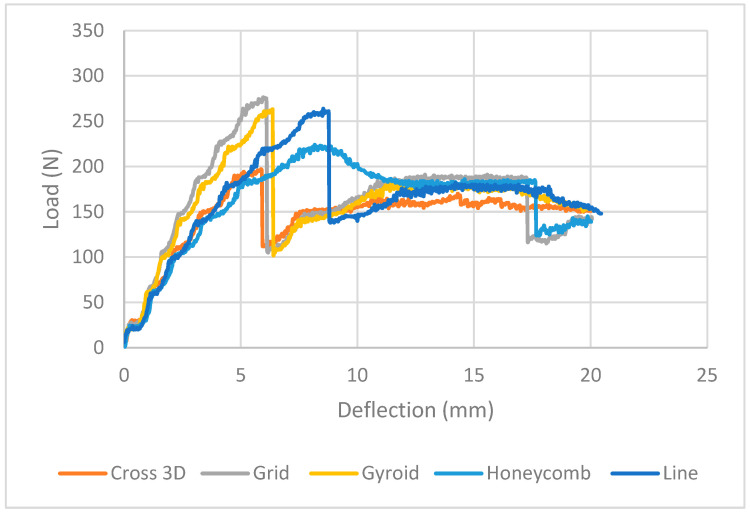
Load vs. Extension PLA-TPU-PLA, (Experimental) Full Zone.

In the elastic range (0–3 mm) the stiffer (steepest slope) is the Grid core in Figure 10 (Table 5). The Grid infill had the best ultimate tensile strength at 20% infill; this is better than other infill patterns and its line filaments are aligned with the tensile axis, and the unsupported spans are also minimized, thus, fewer local instabilities are found than the thin honeycomb walls of the same mass [29]. Then, Grid ≈ Line, then Cross-3D, with Honeycomb softest. Optimal loads are at Grid 270–280 N (5.5–6 mm), Gyroid 1 255–265 N (108 mm) and Line 255–265 N (108 mm), Honeycomb 215–220 N (6 mm), and Cross-3D 185–195 N (6 mm). It is these curves, which tend to be suddenly dipped Cross-3D (~6 mm), Line (8.7 mm), Honeycomb ~17.5 mm), Grid (17.5 mm), to local rib buckling, face-sheet cracking or skin-core debonding, after which all the patterns then proceed to undergo a lengthy plateau. Gyroid and Line have the highest residual loads (up to 20 mm) with maximum energy absorption and maximum stability of softening; Honeycomb lower and smoother (up to 130–150 N); and Grid gives maximum softening after the peak (up to 120–140 N), although this is a high stiffness/strength material.

**Figure 11:**
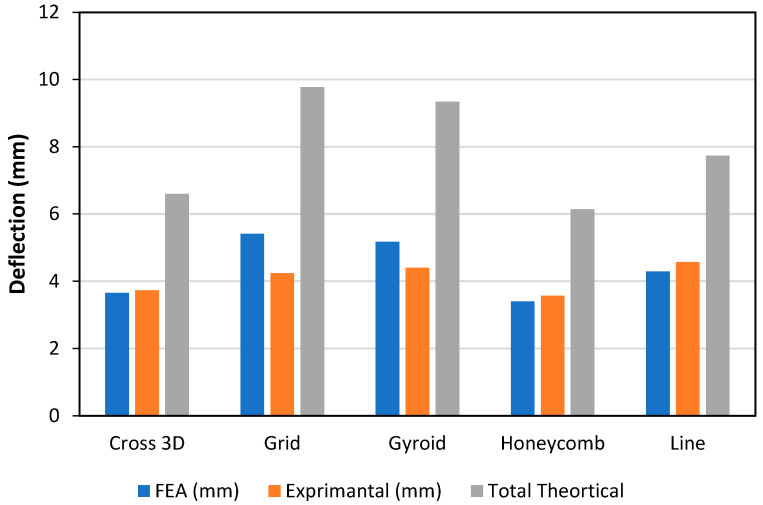
Deflection and Infill Pattern, PLA-TPU-PLA (Elastic Zone).

Overall, all patterns showed the classical theory provides the largest deflections (i.e., over-predicts compliance by ~2–5 mm) with the largest difference for Grid and Gyroid (≈~9.8 and ~9.3 mm versus tests ~ 4.2–4.3 mm) as depicted in Figure 11. FEA was much closer to the tests (typically ~0.3–1.2 mm): Cross-3D ≈ 3.6–3.7 mm, Honeycomb ≈ 3.3–3.6 mm, Grid ≈ 5.4 versus 4.2 mm, Gyroid ≈ 5.1 versus 4.3 mm, Line ≈ 4.2 versus 4.6 mm. The experimental ranking of stiffness (lower deflection = stiffer) is Honeycomb ≈ Cross-3D (Stiffest) → Grid ≈ Gyroid → Line (most compliant). FEA has a reasonable reproduction of this ranking, while theory tends to exaggerate the differences, as well as to generally predict too-soft cores. The bias in the theory is likely due to homogenized shear assumptions and omitting TPU nonlinear strain hardening and rib-junction restraint; remaining FEA—test differences are commensurate with skin–core slip and print variability.

With identical TPU cores and infill patterns, the load-deflection response is governed by the face material. PA face has the highest capacity load and stiffness, while PET one experienced more deflection. PLA face sheets produced the lowest peak loads and largest deflections, reflecting reduced bending resistance under the same structural configuration.

### 5.2. Comparing Non-Interlock with Interlock Composites (Cross 3D Pattern)

**Figure 12:**
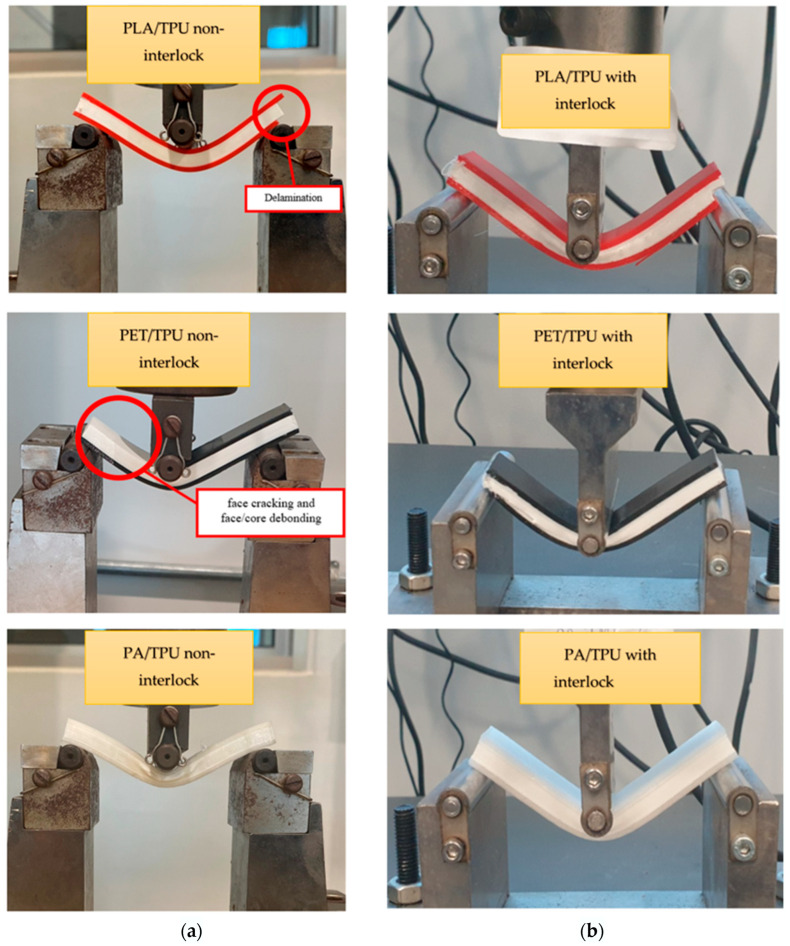
(**a**) non-interlocked structure; (**b**) interlocked structure.

All samples with interlock features were tested under three-point bending and compared with specimens fabricated without interlock features. Mechanical properties were assessed for both interlocked and non-interlocked specimens, including maximum deflection, maximum force, and energy absorption, to evaluate failure modes and deformation behavior. Figure 12 shows the common mode panel failure bending for both configurations. In the non-interlocked specimens, deformation primarily took place in the upper section, where they experienced compressive stress beneath the loading nose, which failed by local fracture and peel-out, resulting in delamination and detachment of the skin from the core, as opposed to the fracture failure mechanism observed in the other core structures [30]. In this section, since the Cross-3D pattern exhibited the lowest deflection among the non-interlocked samples, we selected it for further investigation to incorporate the interlocking feature into the composite structures.

**Figure 13:**
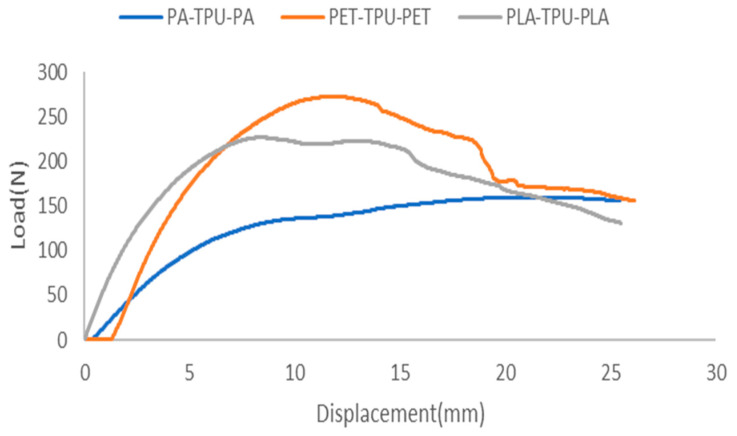
Force vs. deflection of interlocked composites.

Figure 13 illustrates the force vs. deflection response of the interlocked structures. Initially, the deformation behavior of all sandwich specimens is linear, indicating elastic deformation due to core compression and bending of the upper face sheet. Once the yield strengths of both the face sheet and the core are reached, the structure transitions into the plastic region.

**Figure 14:**
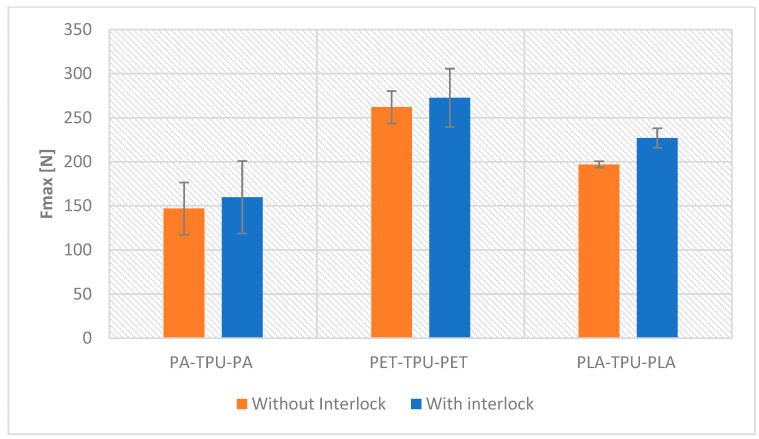
The peak load analysis of different systems.

As shown in Figure 14, the load-deflection profiles reveal that the impact of the interlocking feature varies with the skin material. A measurable enhancement in load capacity was observed for all interlocked designs compared to non-interlocked ones. Among all configurations, the PET/TPU exhibited the lowest gain in peak load, increasing from 261.9 ± 18 N to 272.6 ± 33 N, a relative gain of 4%m, since its initial adhesion is already strong and the interlock simply supplements load transfer, while PA/TPU increased from 146.9 ± 29 N to 159.8 ± 41 N (+8.7%). PLA/TPU achieved the most significant load gain, rising from 196.9 ± 3 N to 227 ± 11 N, indicating that stiff PLA benefits most from interlocking. For the PA–TPU and PET–TPU sandwich structures, the peak load differences between interlocked and non-interlocked specimens fall within experimental scatter and are not statistically significant. These results align with values reported in previous studies. As an example, Ahmed et al. [31] found that 3D printed PLA/TPU sandwich beams sustained a maximum load of 260 N under bending. Such a performance trend aligns with prior research indicating that interlocks improve the load response. As reported by Zhu et al. [32] orthogrid interlocking designs provided higher stiffness and uniform load transfer than standard cores.

**Figure 15:**
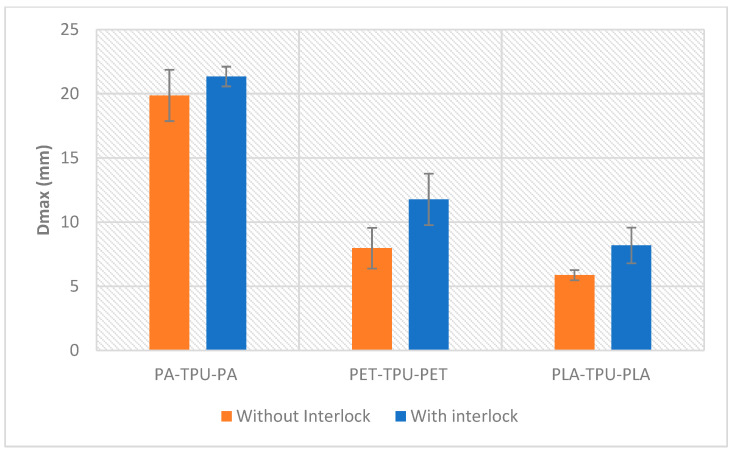
Maximum delfection of different systems.

As depicted in Figure 15, the peak deflection values indicate that interlocked models could flex further and undergo greater bending before failure compared to conventional sandwich ones. For instance, PET/TPU samples had the most significant gain, increasing from 7.96 ± 1.5 mm to 11.77 ± 2 mm (+47.9%) indicating effective engagement of the compliant TPU core. The maximum displacement of PLA/TPU increased from 5.87 ± 0.4 mm to 8.18 ± 0.065 mm (+39.4%). The lowest increase was observed for PA/TPU, which grew slightly from 19.87 ± 2 mm to 21.34 ± 0.7 mm (+7.4%). The PA-TPU-PA composite is the most flexible and withstands the greatest deflection before failure indicates that PA has the highest ductility and toughness. The stiffness of the PLA faces is of a more brittle nature, giving the greatest flexural rigidity and the least amount of deflection. PET has an intermediate modulus and ductility. In a study by Gohar et al. it was observed that the worst performance of PLA/TPU parts because of interfacial adhesion strength, reflecting that poor face core adhesion is the main limiting factor for PLA-TPU sandwich deflection and strength [33].

Similarly, Jiang et al. found that interlocked CFRP orthogrids sustained more deformation and ductility before failure than non-interlocked trusses [34]. In line with our study, Ahmed et al. demonstrated that sandwich beams with glass-fiber-reinforced polyamide (PA12) skins and TPU cores, using gyroid and octet infill topologies, reached central deflections of up to ~33 mm under flexural loading at 260 N without interfacial failure [31].

The energy absorption is represented by the area under the stress–strain profiles and it was calculated until a 20% strain was reached, corresponding to the onset of core densification observed in preliminary tests, hence energy absorption is reported up to this strain [35]. Although this quantity is not identical to the total energy absorbed by the sandwich, it can be regarded as a representative measure of energy absorption.

**Figure 16:**
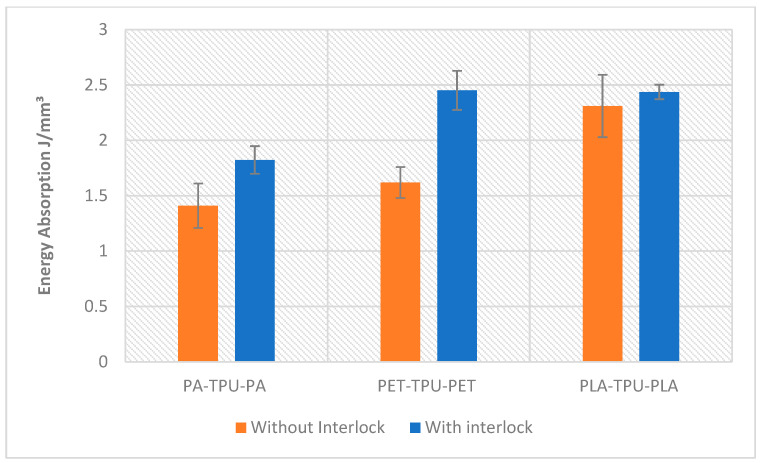
Energy absorption analysis of all specimens.

Figure 16 illustrates the evaluation of energy absorption for 3D-printed multilayers under bending. The largest improvement was observed in the PET/TPU structure, increasing from 1.62 ± 0.14 to 2.45 ± 0.17 J/mm^3^ (+51.4%). PA/TPU-PA showed a moderate rise from 1.41 ± 0.2 to 1.82 ± 0.12 J/mm^3^ (+29.3, while PLA/TPU-PLA exhibited only a slight increase from 2.31 ± 0.28 to 2.44 ± 0.065 J/mm^3^ (+5.5%). This slight difference implies that the PLA skins likely undergo brittle fracture early, limiting additional energy uptake even when interface bonding is reinforced. On the other hand, the PET/TPU interface was slightly less bonded, but had more energy absorption attributable to plastic deformation which suggests that the interlocks work effectively to enhance stress transfer to avoid skin–core debonding.

Overall, the results show that the interlocked configuration exhibited the highest effectiveness, most notably observed in PET reinforced composites, where the energy absorption capacity increased by more than 50% relative to the baseline. Interlock performance is a function of skin load capacity-to-ductility ratio and skin/TPU adhesive properties. Engineered interlocks act as shear-transfer connectors that prevent delamination, promote skin/core synergetic deformation and increase total energy absorbed due to stable progressive failure.

## 6. Conclusions

Regardless of interlocking, the bending performance of the sandwich panels was highly dependent on the arrangement of internal infill. In the current work, grid and gyroid geometries offered the highest load and stiffness with an expansion-dominated deformation response while line offered a middle stiffness–ductility response. Cross-3D and Honeycomb cores had lower strength.

The Cross-3D had the lowest deflection among the non-interlocked specimens; this was used to assess whether the contribution of the slicer-made interfacial interlocks was an interlocking evaluation. It is evident that the interfacial interlocks of 3D printed sandwich panels have impressive re-interlocking because when it comes to flexural properties, the combination of energy absorption, pre-failure displacement and total load effectiveness suggests so. Relative to the materials assessed, a greater level of energy absorption and load bearing capacity was achieved with the PET/TPU interlocked configuration while a more significant capacity of deformation occurred with the PA/TPU interlocked laminate. However, the PET/TPU interlocked provided the optimal balance between different flexural characteristics, which substantiates the utilization of slicer based interlocks for structural integrity. Such a design could be used where energy absorption and manufacturability are priorities in automotive impact scenarios, aerospace applications and lightweight civil engineering applications.

Because the present study was limited to flexural characterization and the Cross-3D infill structure, future efforts should be more expansive in correlating different architectures—Grid, Gyroid, Honeycomb infills—and clearer topology to interface on the exterior to determine performance parameters. The same holds true for impact, fatigue and multi-axial loading stress testing that would improve mechanical design considerations for slicer based interlocking composites.

## Figures and Tables

**Table 1:** Characteristics of the 3D-printed sandwich composite.

Composite	Skin Material (Elastic Modulus, Solid) [AA]	Core Material (Effective Elastic Modulus, 20% Infill Density) [Equation (9)]
PET/TPU	PET (1933 MPa)	TPU (8.2 MPa)
PA/TPU	PA (2419 MPa)	
PLA/TPU	PLA (2308 MPa)	

**Table 2:** Printing parameters. Adapted from [19], Polymers, 2020.

Parameters Used	Settings
Printing speed	1.167 mm/s
Temperature of the printing	205 °C
Temperature of the printing bed	65 °C
Height of the printed layer	0.2 mm
Thickness of the wall	1 mm
Thickness of the top layer	1 mm
Thickness of the bottom layer	1 mm

**Table 3:** PA-TPU-PA Patterns Details.

Infill Pattern	Peak Load (N)	Deflection at Peak (mm)	Post-Peak Plateau (N)	Notes
Grid	220–230	9–11	175–185	Highest capacity; steady after initial crest.
Line	180–185	11–14	170–180 (minor dip near ~19 mm)	Long, flat shoulder → strong energy area.
Gyroid	185–190	9–11	170–175	Smooth crest, gentle decline.
Cross-3D	140–145	9–12	140–145	Lower but very consistent load.
Honeycomb	110–115	8–10	100–105	Softest overall; for low-load uses.

**Table 4:** PET-TPU-PET.

Infill Pattern	Peak Load (N)	Deflection at Peak (mm)	Plateau Near 20 mm (N)	Behavior Note
Grid	350	9–10	295–300	Highest crest and sustained load
Gyroid	325–330	8–10	270–280	Smooth crest, stable tail
Line	300–310	10–12	230–240	Longer shoulder, more softening
Cross-3D	255–265	8–9	235–245	Mid/low capacity, steady
Honeycomb	220–225	8–9	175–185	Softest; early wall bending

**Table 5:** PLA-TPU-PLA, 20%, All Patterns Details.

Infill Pattern	Peak Load (N)/First Drop (N at mm)	Plateau Level at 15–20 mm (N)	Ductility/Stability (Qual.)	Notes/Use-Case
Grid	270–280 at 5.5–6	120–140	Moderate softening after peak	Best for high initial stiffness; largest post-peak reduction.
Gyroid	255–265 at 8–9	150–170	Stable plateau, good spread	Good energy absorption with high residual load.
Line	255–265 at 8–9	150–165	Stable plateau, mild softening	Balanced stiffness/ductility; strong large-deflection support.
Honeycomb	215–220 at 8–9	130–150	Smooth response, no sharp drops	Most compliant; predictable but lower strength.
Cross-3D	185–195 at 6 (local drop near 6 mm)	150–160	Plateau stable after early event	Early local failure then steady carry; decent residual load.

## Data Availability

The original contributions presented in this study are included in the article. Further inquiries can be directed to the corresponding author.
